# The burden of disease and the cost of illness attributable to child maltreatment in Japan: long-term health consequences largely matter

**DOI:** 10.1186/s12889-020-09397-8

**Published:** 2020-08-27

**Authors:** Xiuting Mo, Ruoyan Tobe Gai, Yoshiyuki Tachibana, Timothy Bolt, Yoshimitsu Takahashi, Takeo Nakayama

**Affiliations:** 1 Department of Health Policy, National Center for Child Health and Development, Tokyo, Japan; 2 Department of Health Informatics, Kyoto University School of Public Health, Kyoto, Japan; 3 Department of Empirical Social Security Research, National Institute of Population and Social Security Research, Uchisaiwaicho 2-2-3, Chiyoda-ku, Tokyo, 1000011 Japan; 4 Maternal-Child Psychiatry, Department of Psychosocial Medicine, National Center for Child Health and Development, Tokyo, Japan; 5 Faculty of Economics, Saitama University, Sakura-ku, Japan

**Keywords:** Child maltreatment, Burden of disease study, Lifelong health consequences, Disability-adjusted life year (DALY), Cost-of-illness

## Abstract

**Background:**

Child maltreatment leads to enormous adverse short- and long-term health outcomes. The aim of this study is to estimate the burden of disease and the cost of illness attributable to child maltreatment in Japan.

**Methods:**

An incidence-based top-down cost of illness analysis was conducted to estimate medical costs and burden of disease attributable to child maltreatment based on a societal perspective. The assessment included short-term and long-term medical costs and burden of disease measured by Disability-Adjusted Life Years (DALYs) that generates mortality and morbidities, based on several national surveys and systematic review. We considered the main types of child maltreatment as exposure, for which the incidence was obtained from literature review. Based on population attributable fractions (PAFs), burden of disease of physical and mental health consequences attributable to child maltreatment were estimated. Then DALYs were converted into monetary value. The lifetime economic burden was finally estimated by combining with medical costs and subject to sensitivity analysis.

**Results:**

The lifetime disease burden expressed in DALYs was estimated at 1,047,580 DALYs (95% CI 788,388 - 1326,80 DALYs) for the cohort victims in 2016. Based on the incidence according to literature review, the overall lifetime economic burden was 50.24 billion USD, equivalent to 1.3 million times of gross domestic product (GDP) per capita. Among the total economic burden, costs of suffering and pain based on DALYs were accounting for 81.3%. These estimates were 7–8 times of conservative estimates which used incidence data from official reported cases.

**Conclusions:**

This study found that the national lifetime cost was huge and equivalent to 1.3 million GDP per capita, and its burden of disease was approximately equal to that of colon and rectum cancers or stomach cancer. Our findings particularly in terms of revealed the considerable burden of disease in long term and potential effects of the strengthened maternal and child care as the preventive strategy.

## Background

Child maltreatment is a raising concern in public health and social welfare in Japan [1] The reported number of suspected cases of child maltreatment is increasing, from 37,323 in 2006 to 122,575 in 2016 [2]. According to the Ministry of Health, Labour and Welfare (MHLW) of Japan, child maltreatment is categorised into four essential types, physical, sexual or psychological (including witnessing domestic violence, WDV) abuse and neglect [3]. Exposure to multiple types and repeated episodes of maltreatment during childhood is associated with high risks to enormous adverse health outcomes, causing a significant social and economic burden on individuals, families, and societies. Those adverse outcomes during childhood include child death, injuries and disabilities, developmental and behavioural problems. Moreover, the related physical and mental health conditions persist into adulthood, leading to the onset of chronic diseases, depression, drug, alcohol misuse and risk sexual behaviour, suicide ideation [4, 5].

The government has introduced a couple of protective measures, with increasing public budget [6–8]. Assessment of costs and burden of disease helps development of resource allocation and priority setting in public sector. Paralleling with growing concerns on child maltreatment, the number of the related analysis of the prevalence, health consequences and economic burden is increasing. So far, for the economic burden there are two typical research frameworks: one is a comprehensively costs evaluation from healthcare, social, educational areas, and loss in productivity et al. [9–13]; another one is to measure related economic and disease burden [14, 15]. Wada et al. (2014) reported the social costs of child abuse in Japan included direct costs of dealing with abuse and the indirect costs related to long-term damage from abuse during the fiscal year 2012. On the other hand, the first framework is likely to underestimate long-term deleterious effects of child maltreatment, on which evidence derived from longitudinal studies is less available compared to that on the short-term counterpart [16]. By integrating previous evidence, our cost-of-illness study aimed to assess lifetime economic and disease burden of mortality and morbidities attributed to child maltreatment based on the later framework, in order to address the evidence gap. We extended cost calculations for monetary values converted from Disability Adjusted Life Years (DALYs), covering related mortality and morbidities [17].

## Methods

An incidence-based (victims estimated by incidence), top-down approach (or attributable risk approach, measuring the proportion of a disease that is due to exposure to risk factor) was applied in this study from a societal perspective [18]. We employed the following steps to estimate the total economic burden, constituted by direct and indirect costs:

1. Population attributable fraction (PAF) was generated to estimate long-term impacts/costs attributed to child maltreatment.
2. Short-term and long-term direct medical costs were assessed by using national expenditure databases.
3. Indirect costs measured include productivity loss caused by abusive head trauma and economic burden deriving from DALYs.
4. Finally, sensitivity analyses were performed for the plausible range of the discount rate and the incidence / prevalence.

### Estimating PAF

In the top-down approach, PAF for each disease *i* measured that how health outcomes and their associated costs may be attributed to child abuse, using the following formula [19, 20]:

$$ PAFi=\frac{P\left({RR}_i-1\right)}{P\left({RR}_i-1\right)+1} $$

(*P:* prevalence of child abuse; *RR:* the relative risk of the outcome *i* in those who experienced child abuse compared with those who did not)

#### Risk ratio (RR) or odds ratio (OR)

Several previous related systematic reviews and meta-analyses summarised the relevant health consequences [5, 15, 21, 22]. As adverse childhood experiences (ACEs) often intertwine with child maltreatment, cluster in children’s lives and cumulatively lead to poor health outcomes, we pooled the ORs from a recent systematic review and meta-analysis for the effect of multiple ACEs on health [5], rather than that for each category of child maltreatment.

#### The pooled prevalence

A literature review was performed to synthesize the evidence on epidemiological characteristics the consequences in Japan. The review focused on those published between December 2010 and March 2018 on Medline (PubMed), Web of Science, SCOPUS and CiNii Articles (Japanese literature). Details of the search strategy, search terms used, and inclusion and exclusion criteria are provided in the Additional file 2. We combined our review results with those studies in Japan included in an existing systematic review [23], and calculated the simple size-weighted mean incidence/prevalence. In addition, the median value was also calculated to examine the robustness (Supplementary Table 1). The annual incidence rate was obtained by the formula: [24].

$$ \mathrm{Incidence}\ \mathrm{rate}=\frac{\mathrm{Prevalence}}{\mathrm{Average}\ \mathrm{duration}} $$

Due to the lack of local data on the average duration, we adopted that published in Australia [12]: the average 5.6 years for physical abuse and 2.9 years for sexual abuse. Based on this finding, the weighted average of 4.6 years was used for other categories of abuse.

### Direct medical costs

#### Short-term medical costs

For abusive head trauma (AHT) is the leading cause of death due to child abuse among children younger than 5 years old, [25] we estimated its hospitalization costs as short-term medical costs by multiplying the incidence of AHT under 3 years old [26], the age-specific population in 2016 [27], and admission medical fee per case [26].

There were two reported incidences: one is the “possible” incidence considering countable possibility of AHT cases at most and another one is the “presumptive” incidence representing victims had intracranial injuries or intentional injuries with certain ICD-10 code. We used the “possible” incidence for the general calculation and the latter one in sensitivity analysis. The total possible AHT cases aged under 3 years was about 8 times of the presumptive counterparts [26].

#### Long-term medical costs

For long-term medical costs, we used National healthcare expenditures 2016 and Patient Survey 2014 to simulate disease burden of relevant health consequences by sex and age group (0–14, 14–44, 45–64, above 65), and then multiplied with PAFs to calculate the attributable costs in the victim cohort of 2016 [5, 26, 28]. On the other hand, we did not include self-harm and collective violence because of the limitation to distinguish the two in the reported overall injury cases.

### Indirect costs

In this study, we considered differential and loss of earning as a result of human capital depreciation is caused by mortality and morbidities. It was presented as a monetary value of DALYs and GDP per capita [15, 29]..

#### DALYs and its monetary value

The disease burden indicator DALY aggregates years of life lost for premature death and years lost due to disability for morbidities [19]. Related data were obtained from the WHO Global Burden of Disease (GBD) [30]. Using the pooled ORs as described by Karen et al. (2017) [5], we matched each related health outcome [5] with the cause of disease burden in the WHO GBD categories, though it was difficult to match some outcomes with the cause of GBD (Supplementary Table 2).

Then, monetary value was converted from DALY attributable to child maltreatment by multiplying DALY and GDP per capita [17] with adjustment of purchasing power parity in 2016 [31].

#### Productivity losses due to AHT fatal cases

Productivity loss due to fatal cases of child maltreatment was calculated based on the reported fatal cases, which figure was obtained from official data [32], and the average lifetime income subject to discounting. In 2016, there were 67 abuse-related deaths reported in Japan (not including family suicide), with the average onset age of 2 years [32]. The discounted lifetime income (from 18 to 65 years old) was calculated by assuming the long-term growth in labour productivity to be 1% per year [10].

#### DALYs losses of survival AHT

For disease burden due to survival AHT, we considered sequelae such as vision loss, brain damage, and reduced life span [33], and long-term health consequences as developing diseases in adulthood. We calculated the disease burden of AHT in 2016 by multiplying average cases and the estimated mean lifetime DALY loss per case at different severity (mild, moderate and severe) [33].

#### Long-term DALYs losses of other diseases

Then the long-term health consequences were calculated using the following formula:

$$ {\displaystyle \begin{array}{l}\mathrm{DALY}\ \mathrm{losses}=\Sigma \left({\mathrm{PAF}}_{\mathrm{i}}\ast {\mathrm{original}\ \mathrm{DALY}}_{\mathrm{i}}\right)\\ {}\left(\mathrm{i}:\mathrm{different}\ \mathrm{child}\ \mathrm{abuse}-\mathrm{related}\ \mathrm{health}\ \mathrm{outcome}\right)\end{array}} $$

### Sensitivity analyses

A discount rate of 2% is generally performed which was recommended in the domestic guideline for cost-effectiveness analysis [34]. Whereas especially in the US, a discount rate of 3% has been selected and applied in the cost estimate reports of Centers for Disease Control and a best practices for the Social Return on Investment analysis recommended by experts and guidelines [9]. As such the parameter potentially affects the finally results, we adopted a plausible range of 0, 2 to 3% for sensitivity analysis.

In addition, we also calculated costs and disease burden using the incidence/prevalence data based on officially reported child abuse cases. To calculate the conservative incidence of child abuse by categories, we obtained the official data of victim cases reported by child consultation facilities in 2016 [2] and then divided them by the total population number in corresponding age [27]. Data by sex were not available. Co-current information was not available and the overlapped cases were not considered (Supplementary Table 3). The initial victim age is assumed to be 7 years old, according to an age-weighted incidence calculation based on official reported cases [2]. We assumed the probable abuse-related death cases to be 8 times of the reported cases based on the ratio of the presumptive and the possible incidence of AHT cases among children aged under 3 years [26].

## Results

The main results showed in tables were discounted at 2% and conservative estimates were given for sensitivity analyses.

### The pooled incidence / prevalence and disease burden

The estimations on different types of child maltreatment incidence draw from literature reviews varied. Regarding differences between sex, except physical abuse, girls suffered more than boys in sexual abuse and witnessing domestic violence (Table 1). The estimated lifetime disease burden associated with child maltreatment onset in 2016 was considerable, 1,047,580 DALYs with a 95% CI of 788,388 DALYs to 1326,806 DALYs (Table 2). The top causes of total disease burden due to child abuse were suicide attempts (34%), cancer (22%), cardiovascular disease (13%) and depression (9%).

**Table 1:** Estimated incidence/prevalence of child abuse in Japan

Estimates ^a^	Prevalence		Incidence ^b^	
	Male	Female	Male	Female
Physical abuse (%)	8.40	6.69	1.50	1.19
Sexual abuse (%)	0.33	7.38	0.11	2.54
Psychological abuse				
WDV ^c^ (%)	9.94	16.75	2.16	3.64
Other ^d^ (%)	5.64	5.95	1.23	1.29
Neglect (%)	8.89	9.01	1.93	1.96

^a^ sample sized weighted mean value

^b^ Incidence rate = Prevalence / Average duration

^c^ WDV: witnessing domestic violence

^d^ not specified as WDV, often expressed as emotional/psychological abuse

**Table 2:** long-term DALY lost attributable to child abuse in Japan

Diseases attributed to child abuse ^**a**^	DALYs	95% Confidence interval	
Suicide attempt	356,773	300,916	399,629
Cancer	229,020	149,110	324,600
Cardiovascular disease	136,215	86,896	194,281
Depression	96,998	79,403	116,032
Respiratory disease	79,628	59,650	102,188
Liver or digestive disease	45,286	33,453	58,506
Anxiety	32,436	21,159	45,572
Problematic drug use	25,410	20,857	30,003
Abusive head trauma	18,504	18,504	18,549
Problematic alcohol use	10,002	8082	12,059
Diabetes	8072	4314	12,512
Sexually transmitted infections	5023	2717	7728
Violence victimisation	3865	3054	4721
Violence perpetration	348	272	427
Total	1,047,580	788,388	1326,806
DALYs monetary value (billion, US$)	40.84	30.73	51.72

^a^ simple size weighted mean prevalence at 2% discounted rate

### Cost-of-illness analysis for child maltreatment

Table 3 demonstrates lifetime costs attributed to child abuse onset in 2016. The total direct cost was estimated to be USD 8930.6 million (95%CI: 6502.1 million-11,674.4 million) while the total indirect cost was estimated to be USD 41,305.7 million (95%CI: 30,965.5 million-52,429.2 million), accounting for 82.2% of the total lifetime costs, which were almost 1.3 million times GDP per capita. Economic loss initiated from DALYs in long-term (costs of suffering and pain) accounted for 81.3% of the overall estimates.

**Table 3:** Lifetime costs attributable to child abuse for the first time in 2016

Items of the costs (USD, million)		95% CI		%
**Direct costs (Medical costs)**	8930.6	6502.1	11,674.4	17.8%
Short-term (AHT)	4.9	3.8	6.0	0.0%
Long-term (other diseases)	8925.7	6498.3	11,668.5	17.8%
**Indirect costs**	41,305.7	30,965.5	52,429.2	82.2%
Abuse death ^a^ (Productivity losses)	468.2	234.1	702.3	0.9%
Survival AHT (DALYs) ^b^	721.0	719.0	727.0	1.4%
Long-term loss of other diseases ^b^	40,116.4	30,012.4	50,999.8	79.9%
**Total costs**	50,236.3	37,467.7	64,103.6	1.3 million GDP per capita

*AHT* Abusive head trauma

^a^: we used 0.5–1.5 times of base line data for range costs of child abuse

^b^: Costs of suffering and pain: DALYs converted into monetary value by multiplying a gross domestic product per capita

### Sensitivity analyses

Conservative estimates based on the reported cases incidence showed a tendency similar to that observed in the disease burden based on the literature review, among which psychological abuse (including WDV) accounted for the majority of reported child abuse cases (51.5%). However, the incidence estimated from the review were much higher than those reported by child protection agencies (the conservative estimation), leading to about 7–8 times difference gap on child maltreat burden by difference discount rate (0, 2, 3%). (Table 4).

**Table 4:** Sensitivity analyses on incidence resource and discounted rate

Sensitivity Analysis	Disease burden in DALYs (95%CI)			Economic burden (USD, million) (95%CI)		
**Literature based estimation** ^**a**^						
dr = 3%	687,769	524,469	861,008	33,194.78	25,024.37	41,959.60
dr = 2%	1,047,580	788,388	1326,806	50,236.3	37,467.7	64,103.6
dr = 0%	2,784,035	2,041,484	3,605,376	132,634.80	96,800.53	172,400.50
**Conservative estimation** ^**b**^						
dr = 3%	99,131	64,477	146,048	4434.64	2897.38	6495.84
dr = 2%	143,520	93,294	211,257	6426.83	4197.12	9408.04
dr = 0%	341,670	222,038	501,550	15,425.35	10,071.83	22,528.25

*dr* discounted rate

^a^ Estimated based on literature review, simple size weighted average prevalence

^b^ Estimated based on the number of consultation cases disposed about child abuse at child guidance centres. Probable estimate of abuse death was assumed about 8 times (confirmed AHT cases/possible cases = 8) of the costs of conservative estimate

## Discussion

Our results indicated that disease and economic burden attributable to child maltreatment is substantial. In particular, that originated from the long-term health consequences accounts for the majority.

Based on literature review, the pooled incidence of child maltreatment in Japan is much higher than officially reported, which is consistent with the findings of other studies [10, 12, 35]. Because of difficulty to identify the actual cases and a public attitude to consider child abuse as a private affair in the society, the officially reported cases are likely to represent the tip of an iceberg.

Psychological abuse (including WDV) represented the majority of reported cases. The results of the literature review also showed a gender difference in the prevalence of the four types of child abuse (size weighted mean values), girls were found to be more likely to experience the harmful practices compared to boys, particularly sexual abuse. This tendency was also observed in other countries in East Asia and Pacific region [15]. Comparing those living in other countries in the East Asia and Pacific region [15, 23], Japanese children tended to less likely to experience physical abuse (boys: 8.40% vs. 16.76%; girls: 6.69% vs. 15.42%).

Although it is difficult to directly compare the results across different study settings due to the different methodologies, parameters and target populations adopted, the ingredients of the lifetime economic and disease burden considered in our study, including medical costs and monetary value of disease burden, are similar to that adopted in previous studies [10, 12]. Still our results showed that the disease burden was about 7–8 times of the conservative estimation due to the huge gap of incidence generated from literature and that officially reported. The number is consistent with an Australian research that showed a wide distribution of the annual prevalence, ranging from 0.85 to 4.6% [12]. In the conservative lifetime course simulation, the initial victim age is assumed to be 7 years old according to an age-weighted incidence calculation based on official reported cases, which was also consistent with previous studies [9].

Our study in particular highlighted DALYs in long-term attributable to child maltreatment, accounting for a relevant proportion (81.3%) in the overall lifetime costs. The estimation of disease burden attributed to child maltreatment (1,047,580 DALYs) was comparable to the total DALYs due to colon and rectum cancers (1,043,335 DALYs in 2015), or stomach cancer (1,002,252 DALYs in 2015) [30].

To our knowledge, this is the first study to estimate lifetime economic burden of child maltreatment in Japan based on an epidemiological model. The idea of this method is to convert disease-induced losses of well-being into economic terms by multiplying the annual number of lost life years due to disease by sub-reginal per capita income. So far, few studies had ever taken this part of costs into account, potentially leading to an underestimation of health and economic impacts of child maltreatment. In addition, we adopted conservative calculation methodology in the sensitivity analyses to estimate the burden of child maltreatment for more reliable range estimations.

There are several limitations to this study. *First*, the co-occurrence of multiple types of child abuse is prevalent [35], resulting in difficulties to identify the adverse effects separately. In order to minimize possible consequent overestimation, we used the pooled ORs of multiple adverse childhood health experiences instead of each types of child maltreatment and its severity. *Second*, we focused on the economic burden due to the mortality and morbidity of child maltreatment but did not consider non-health human capital aspects, to address the knowledge gap. *Third*, like other economic burden estimation studies, the availability of data on the related medical costs were limited. We nevertheless targeted major health consequences and explored their unit costs for the estimates [26].

Recently in Japan, a continuum of intensive supports to mothers and child-rearing families encompassing the reproductive cycle has been widely implemented in most local authorities. Such an integral approach serves as an essential preventive strategy against child maltreatment and other harmful practices by early detection and intervention of high-risk households in pregnancy, postpartum and child-rearing periods. This study can provide decision makers information on the economic burden of child maltreatment, as well as an important input in future economic evaluations (cost-effectiveness analysis) on currently ongoing intervention and policy. In addition, our results hint an emphasis on preventive interventions on suicide attempts and depression, which are top causes of the attributable disease burden due to child maltreatment.

## Conclusion

Our study demonstrated that lifetime disease and economic burden due to child maltreatment in Japan is substantial. Its disease burden was approximately equal to the burden of colon and rectum cancers or stomach cancer. In particular, it is important to include the long-term disease burden in future studies related to disease burden and cost of illness for both technical and policy perspectives.

## Supplementary information

**Additional file 1 Table a1.** Studies included in the quantitative synthesis. **Table a2.** Health outcomes and pooled ORs used in this study (AHT not included). **Table a3.** Incidence rate by age and average onset age, based on the number of consultation cases disposed about child abuse at child guidance centers.

**Additional file 2 **Systematics review (2018/5/10–2018/5/20)-find possible literature including Japanese studies on risk of health outcomes attributable to child maltreatment. **Figure a1.** Study selection (PRISMA) flow diagram.

## Data Availability

All the raw data is publicly accessible from respective official website as reference (National healthcare expenditures 2016 and Patient Survey 2014). The datasets analysed during the current study are available from the corresponding author on reasonable request.

## Abbreviations

DALYs: Disability-Adjusted Life Years
PAFs: population attributable fractions
GDP: gross domestic product
MHLW: Ministry of Health, Labour and Welfare
WDV: witnessing domestic violence
AHT: abusive head trauma
ICD: International Classification of Diseases
GBD: Global Burden of Disease
RR: risk ratio
OR: odds ratio
ACEs: adverse childhood experiences
