# Supplementary material for: The burden of disease and the cost of illness attributable to child maltreatment in Japan: long-term health consequences largely matter
Source: BMC Public Health. 2020 Aug 27;20:1296. doi: 10.1186/s12889-020-09397-8 (PMC7450590; doi:10.1186/s12889-020-09397-8)
Supplement: Supplementary file 1 — Additional file 1 Table a1. Studies included in the quantitative synthesis. Table a2. Health outcomes and pooled ORs used in this study (AHT not included). Table a3. Incidence rate by age and average onset age, based on the number of consultation cases disposed about child abuse at child guidance centers. [file 12889_2020_9397_MOESM1_ESM.doc]

Table a1

*Studies included in the quantitative synthesis*

|  |  | Males |  |  | Females |  | Reference |
| --- | --- | --- | --- | --- | --- | --- | --- |
| Year | First Author | Prevalence | Simple size |  | Prevalence | Simple size |  |
| Physical Abuse Prevalence Analysis | | (%) |  |  | (%) |  |  |
| 2018 | Erika Obikane | 5.57 | 1776.00 |  | 5.57 | 2016.00 | 1 |
| 2017 | Sakurako Okuzono | 11.53 | 14983.00 |  | 8.04 | 14199.00 | 2 |
| 2016# | Matsuyama Y | 1.40 | 11700.00 |  | 1.40 | 13489.00 | 3 |
| 2015# | Tadayuki Oshima | 3.20 | 250.00 |  | 3.20 | 250.00 | 4 |
| 2015 | Satoshi Tsuboi | 1.80 | 610.00 |  | 3.73 | 804.00 | 5 |
| 2013 | T. Oshio | 34.10 | 1646.00 |  | 34.10 | 1646.00 | 6 |
| 2011 | Fujiwara T | 7.30 | 871.00 |  | 8.60 | 851.00 | 7.1-7.3 |
| 2007 | Masuda A | 5.60 | 831.00 |  | 6.90 | 761.00 | 8 |
| 2007 | Dussich JPJ | 56.60 | 143.00 |  | 58.00 | 216.00 | 9 |
| 2000 | Kitamura T. | 2.20 | 96.00 |  | 3.30 | 124.00 | 10 |
| Simple Size Weighted Prevalence | | 8.40 |  |  | 6.69 |  |  |
| Median |  | 5.59 |  |  | 6.24 |  |  |
| Sexual Abuse Prevalence Analysis | |  |  |  |  |  |  |
| 2015# | Tadayuki Oshima | 0.40 | 250.00 |  | 0.40 | 250.00 | 4 |
| 2015 | Satoshi Tsuboi | 0.00 | 610.00 |  | 1.12 | 804.00 | 5 |
| 2011 | Fujiwara T | 0.10 | 871.00 |  | 0.90 | 851.00 | 7.1-7.3 |
| 2010 | Yoshihama M | - | 0.00 |  | 10.44 | 1371.00 | 11 |
| 2007 | Masuda A | 0.80 | 831.00 |  | 5.00 | 761.00 | 8 |
| 2007 | Uji M | - | 0.00 |  | 26.00 | 532.00 | 12 |
| Simple Size Weighted Prevalence | | 0.33 |  |  | 7.38 |  |  |
| Median |  | 0.25 |  |  | 3.06 |  |  |
| Neglect Prevalence Analysis | |  |  |  |  |  |  |
| 2018 | Erika Obikane | 1.75 | 1776.00 |  | 2.13 | 2016.00 | 1 |
| 2016# | Matsuyama Y | 11.30 | 11700.00 |  | 11.30 | 13489.00 | 3 |
| 2015 | Satoshi Tsuboi | 0.33 | 610.00 |  | 1.24 | 804.00 | 5 |
| 2013 | T. Oshio | 6.40 | 1646.00 |  | 6.40 | 1646.00 | 6 |
| 2011 | Fujiwara T | 1.78 | 871.00 |  | 1.31 | 851.00 | 7.1-7.3 |
| Simple Size Weighted Prevalence | | 8.89 |  |  | 9.01 |  |  |
| Median |  | 1.78 |  |  | 2.13 |  |  |
| Emotional Abuse Prevalence Analysis | |  |  |  |  |  |  |
| 2016# | Matsuyama Y | 5.30 | 11700.00 |  | 5.30 | 13489.00 | 3 |
| 2015# | Tadayuki Oshima | 12.40 | 250.00 |  | 12.40 | 250.00 | 4 |
| 2015 | Satoshi Tsuboi | 1.31 | 610.00 |  | 5.35 | 804.00 | 5 |
| 2007 | Masuda A | 11.60 | 831.00 |  | 16.00 | 761.00 | 8 |
| Simple Size Weighted Prevalence | | 5.64 |  |  | 5.95 |  |  |
| Median | National Epidemiological Survey | 8.45 |  |  | 8.87 |  |  |
| Witnessing DV Prevalence Analysis | |  |  |  |  |  |  |
| 2010 | Fujiwara T | 9.60 | 871.00 |  | 11.40 | 761.00 | 7.1-7.3 |
| 2010 | Yoshihama M | - | 0.00 |  | 18.57 | 851.00 | 11 |
| 2007 | Masuda A | 10.30 | 831.00 |  | 18.60 | 1371.00 | 8 |
| Simple Size Weighted Prevalence | | 9.94 |  |  | 16.75 |  |  |
| Median |  | 9.95 |  |  | 18.57 |  |  |

# gender difference data not available

Reference

| 1 | Obikane, E., Shinozaki, T., Takagi, D., & Kawakami, N. (2018). Impact of childhood abuse on suicide-related behavior: Analysis using marginal structural models. Journal of affective disorders, 234, 224-230. |
| --- | --- |
| 2 | Okuzono, S., Fujiwara, T., Kato, T., & Kawachi, I. (2017). Spanking and subsequent behavioral problems in toddlers: A propensity score-matched, prospective study in Japan. Child Abuse & Neglect, 69, 62-71. |
| 3 | Matsuyama, Y., Fujiwara, T., Aida, J., Watt, R. G., Kondo, N., Yamamoto, T., ... & Osaka, K. (2016). Experience of childhood abuse and later number of remaining teeth in older Japanese: a life‐course study from Japan Gerontological Evaluation Study project. Community dentistry and oral epidemiology, 44(6), 531-539. |
| 4 | Oshima, T., Fukui, H., Watari, J., & Miwa, H. (2015). Childhood abuse history is associated with the development of dyspepsia: a population-based survey in Japan. *Journal of gastroenterology*, *50*(7), 744-750. |
| 5 | Tsuboi S, Yoshida H, Ae R, et al. Prevalence and demographic distribution of adult survivors of child abuse in Japan[J]. Asia Pacific Journal of Public Health, 2015, 27(2): NP2578-NP2586. |
| 6 | Oshio, T., Umeda, M., & Kawakami, N. (2013). Impact of interpersonal adversity in childhood on adult mental health: how much is mediated by social support and socio-economic status in Japan?. Public health, 127(8), 754-760. |
| 7.1 | Fujiwara T, Kawakami N, World Mental Health Japan Survey Group. Association of childhood adversities with the first onset of mental disorders in Japan: results from the World Mental Health Japan, 2002–2004[J]. Journal of psychiatric research, 2011, 45(4): 481-487. |
| 7.2 | Umeda, M., Kawakami, N., Kessler, R. C., & Miller, E. (2015). Childhood adversities and adult use of potentially injurious physical discipline in Japan. Journal of family violence, 30(4), 515-527. |
| 7.3 | Stickley, A., Koyanagi, A., Kawakami, N., & WHO World Mental Health Japan Survey Group. (2015). Childhood adversities and adult‐onset chronic pain: Results from the World Mental Health Survey, Japan. European Journal of Pain, 19(10), 1418-1427. |
| 8 | Masuda, A., Yamanaka, T., Hirakawa, T., Koga, Y., Minomo, R., Munemoto, T. & Tei, C., (2007). Intra- and extra-familial adverse childhood experiences and a history of childhood psychosomatic disorders among Japanese university students. BioPsychoSocial Medicine, 1(9), 1-7. |
| 9 | Dussich, J.P.J. & Maekoya, C. (2007). Physical child harm and bullying-related behaviors: A comparative study in Japan, South Africa, and the United States. International Journal of Offender Therapy and Comparative Criminology, 51(5), 495-509. |
| 10 | Kitamura, T., Kaibori, Y., Takara, N., Oga, H., Yamauchi, K. & Fujihara, S. (2000). Child abuse, other early experiences and depression: I. Edpidemiology of parental loss, child abuse, perceived rearing experiences and early life events among a Japanese community population. Archives of Women’s Mental Health, 3, 47-52. |
| 11 | Yoshihama, M. & Horrocks, J. (2010). Risk of intimate partner violence: Role of childhood sexual abuse and sexual initiation in women in Japan. Children and Youth Services Review, 32, 28-37. |
| 12 | Uji, M., Shono, M., Shikai, N. & Kitamura, T. (2007). Case illustrations of negative sexual experiences among university women in Japan: Victimization disclosure and reactions of the confidant. International Journal of Offender Therapy and Comparative Criminology, 51(2), 227-242. |

Table a2

***Health outcomes and pooled ORs used in this study (AHT not included)***

| Health outcomes ^a^ | WHO GDB | Pooled ORs (95%CI) ^a^ |
| --- | --- | --- |
| Suicide attempt | Self-harm | 37.48 (22.19–63.31) |
| Problematic drug use | Drug use disorders | 10.22 (7.62–13.71) |
| Perpetrator of violence | Collective violence and legal intervention | 8.10 (5.87–11.18) |
| Victims of violence | Interpersonal violence | 7.51 (5.60–10.08) |
| Sexually transmitted infections | STDs excluding HIV + HIV/AIDS | 5.92 (3.21–10.92) |
| Problematic alcohol use | Alcohol use disorders | 6.86 (5.36–8.78) |
| Depression | Depressive disorders | 4.74 (3.88–5.80) |
| Anxiety | Anxiety disorders | 3.70 (2.62–5.22) |
| Respiratory disease | Respiratory diseases | 3.05 (2.47–3.77) |
| Liver or digestive disease | Digestive diseases | 2.76 (2.25–3.38) |
| Cancer | Malignant neoplasms | 2.31 (1.82–2.95) |
| Cardiovascular disease | Cardiovascular diseases | 2.07 (1.66–2.59) |
| Diabetes | Diabetes mellitus | 1.38 (1.20–1.60) |

^a^ Data derived from literature: Hughes K, Bellis MA, Hardcastle KA, Sethi D, Butchart A, Mikton C, Jones L, Dunne MP. The effect of multiple adverse childhood experiences on health: a systematic review and meta-analysis. The Lancet Public Health. 2017;2(8):e356-e66.

Table a3

*Incidence rate by age and average onset age, based on the number of consultation cases disposed about child abuse at child guidance centers*

| Age | In total | Physical abuse | Sexual abuse | Psychological abuse (WDV included) | Psychological abuse (WDV not included) | Witness of violence | Neglect |
| --- | --- | --- | --- | --- | --- | --- | --- |
| 0 | 0.75% | 0.12% | 0.00% | 0.42% | 0.16% | 0.26% | 0.21% |
| 1 | 0.84% | 0.13% | 0.00% | 0.55% | 0.22% | 0.33% | 0.16% |
| 2 | 0.85% | 0.15% | 0.00% | 0.52% | 0.24% | 0.28% | 0.18% |
| 3 | 0.81% | 0.17% | 0.00% | 0.47% | 0.22% | 0.25% | 0.17% |
| 4 | 0.78% | 0.17% | 0.00% | 0.44% | 0.21% | 0.23% | 0.16% |
| 5 | 0.72% | 0.17% | 0.01% | 0.39% | 0.18% | 0.21% | 0.16% |
| 6 | 0.73% | 0.18% | 0.00% | 0.39% | 0.18% | 0.21% | 0.16% |
| 7 | 0.71% | 0.18% | 0.01% | 0.36% | 0.17% | 0.19% | 0.16% |
| 8 | 0.69% | 0.19% | 0.01% | 0.35% | 0.16% | 0.18% | 0.15% |
| 9 | 0.67% | 0.19% | 0.01% | 0.34% | 0.17% | 0.17% | 0.14% |
| 10 | 0.63% | 0.18% | 0.01% | 0.31% | 0.15% | 0.16% | 0.13% |
| 11 | 0.61% | 0.18% | 0.01% | 0.29% | 0.14% | 0.15% | 0.13% |
| 12 | 0.58% | 0.18% | 0.01% | 0.26% | 0.13% | 0.13% | 0.12% |
| 13 | 0.57% | 0.20% | 0.01% | 0.25% | 0.13% | 0.13% | 0.11% |
| 14 | 0.52% | 0.18% | 0.02% | 0.22% | 0.11% | 0.11% | 0.10% |
| 15 | 0.43% | 0.15% | 0.01% | 0.18% | 0.09% | 0.09% | 0.08% |
| 16 | 0.34% | 0.12% | 0.02% | 0.15% | 0.07% | 0.08% | 0.06% |
| 17 | 0.27% | 0.10% | 0.01% | 0.12% | 0.06% | 0.06% | 0.05% |
| 18 | 0.06% | 0.02% | 0.00% | 0.02% | 0.01% | 0.01% | 0.02% |
| Total ^a^ | 0.60% | 0.16% | 0.01% | 0.31% | 0.14% | 0.16% | 0.13% |
| Onset age | 7.33 | 8.55 | 11.80 | 6.74 | 7.03 | 6.48 | 7.06 |

^a^ case overlap was not considered

Refer: number of consultation cases disposed about child abuse at child guidance centers in Japan 2016 (in Japanese)

<https://www.e-stat.go.jp/stat-search/files?page=1&layout=datalist&tstat=000001034573&cycle=8&tclass1=000001108815&tclass2=000001108820&second2=1>
