# Supplementary material for: The burden of disease and the cost of illness attributable to child maltreatment in Japan: long-term health consequences largely matter
Source: BMC Public Health. 2020 Aug 27;20:1296. doi: 10.1186/s12889-020-09397-8 (PMC7450590; doi:10.1186/s12889-020-09397-8)
Supplement: Supplementary file 2 — Additional file 2 Systematics review (2018/5/10–2018/5/20)-find possible literature including Japanese studies on risk of health outcomes attributable to child maltreatment. Figure a1. Study selection (PRISMA) flow diagram. [file 12889_2020_9397_MOESM2_ESM.docx]

**Systematics review (2018/5/10-2018/5/20)-find possible literature including Japanese studies on risk of health outcomes attributable to child maltreatment.**

“Child maltreatment prevalence, incidence and consequences in the east Asia and pacific region, a systematic review of research”, included published paper from 2000- November 2010. Besides the included paper in that systematic review, we did an additional search from December 2010 to March 2018.

We followed the PRISMA statement guidelines for systematic review. A systematic database search from December 2010 up to March 201 was performed on Medline (PubMed), Web of Science, SCOPUS, CiNii Articles (Japanese literature). search term related to: (1) child, (2) maltreatment, and (3) Japan were used.

**Inclusion criteria:**

- primary research on the **costs, prevalence, incidence and consequences** of child maltreatment in Japan
- studies of possible consequences were included if:
  - there were primary research studies that explored the relationship between one of the types of child maltreatment and outcomes in any of the following areas: employment, education, mental health, physical health and health behaviours, aggression, violence, criminality, exposure to further violence and services use (the burden of child maltreatment in the east Asia and pacific region)
  - they included odds ratio (OR) or relative risk (RR) calculations disaggregated by type of maltreatment
  - populations were not sampled on the basis of the presence of the specified outcome since these cannot be used to calculate an RR for that outcome
  - they were retrospective or prospective observational studies.
- published between December 2010 to March 2018
- peer-reviewed and non peer-reviewed journal articles, presentations, dissertations or research reports.
- Research published in English and Japanese.

**Excluded criteria:**

Followed the “systematic review”: In a global systematic review of the impact of child abuse on mental health outcomes, Andrews and colleagues (2004) developed two exclusion criteria that were also applied in this study:

- Prevalence studies with a total sample size of less than 100 were excluded unless they were from underrepresented countries, as samples of less than 100 may not produce reliable prevalence estimates.
- Studies where the population was sampled on the basis of the presence of a specified outcome were excluded. For example, we would exclude a study that sampled suicidal and depressed adolescents in country X to find out if they had experienced abuse and neglect. But we would include a study that sampled youth who were and were not exposed to abuse and neglect about their mental health and potential self-harming behaviour. The reason for excluding sampling based on presence or absence of an outcome variable is that these cannot be used to calculate a relative risk for that outcome (Steiner 1998 as cited in Andrews et al, 2004). Therefore, in this review, case-control studies were only included if the ‘cases’ are individuals exposed to one or more of the child maltreatment variables and ‘controls’ are those who are not exposed (Andrews et al., 2004).

Search strategy

**Electronic databases:** PubMed, Web of science, SCOPUS, and CiNii

**Subject heading and keyword searches**: This review utilised both free text and controlled vocabulary of subject heading and keyword searches to identify articles and grey literature via electronic databases. To provide the broadest coverage of articles, the initial search terms consisted of: child (child, childhood, children) AND type of maltreatment (maltreatment, sexual abuse, physical abuse, exploitation, neglect) AND Japan

**In details:**

- PubMed:

child[mh] AND (maltreatment[majr] OR sexual abuse[majr] OR physical abuse[majr] OR exploitation[majr] OR neglect[majr]) AND Japan

Results: 5

- Web of science

TS = (Child AND (maltreatment OR sexual abuse OR physical abuse OR exploitation OR neglect) AND Japan) AND language: (English)

Index =SCI-EXPANDED, SSCI, A&HCI, CPCI-S, CPCI-SSH, BKCI-S, BKCI-SSH, ESCI, CCR-EXPANDED, IC time span=2010-2018

Results: 86

- SCOPUS

TITLE-ABS-KEY ( child AND abuse AND japan ) AND PUBYEAR > 2009 AND PUBYEAR < 2019

Results: 185

- CiNii

Freewords: child abuse Japan (2010-2018)

Results: 198

**Figure a1. Study selection (PRISMA) flow diagram**
